# Association of congenital cardiovascular malformation and neuropsychiatric phenotypes with 15q11.2 (BP1–BP2) deletion in the UK Biobank

**DOI:** 10.1038/s41431-020-0626-8

**Published:** 2020-04-23

**Authors:** Simon G. Williams, Apostol Nakev, Hui Guo, Simon Frain, Gennadiy Tenin, Anna Liakhovitskaia, Priyanka Saha, James R. Priest, Kathryn E. Hentges, Bernard D. Keavney

**Affiliations:** 1 Division of Cardiovascular Sciences, School of Medical Sciences, Faculty of Biology, Medicine and Health, Manchester Academic Health Science Centre, University of Manchester, Manchester, UK; 2 Division of Population Health, Health Services Research and Primary Care, School of Health Sciences, Faculty of Biology, Medicine and Health, University of Manchester, Manchester, UK; 3 Department of Pediatrics, Stanford University, Stanford, CA USA; 4 Division of Evolution and Genomic Sciences, School of Biological Sciences, Faculty of Biology, Medicine and Health, University of Manchester, Manchester, UK

**Keywords:** Chromosome abnormality, Genome informatics, High-throughput screening, Genetics research, Neurodevelopmental disorders

## Abstract

Deletion of a non-imprinted 500kb genomic region at chromosome 15q11.2, between breakpoints 1 and 2 of the Prader–Willi/Angelman locus (BP1–BP2 deletion), has been associated in previous studies with phenotypes including congenital cardiovascular malformations (CVM). Previous studies investigating association between BP1–BP2 deletion and CVM have tended to recruit cases with rarer and more severe CVM phenotypes; the impact of CVM on relatively unselected population cohorts, anticipated to contain chiefly less severe but commoner CHD phenotypes, is relatively unexplored. More precisely defining the impact of BP1–BP2 deletion on CVM risk could be useful to guide genetic counselling, since the deletion is frequently identified in the neurodevelopmental clinic. Using the UK Biobank (UKB) cohort of ~500,000 individuals, we identified individuals with CVM and investigated the association with deletions at the BP1–BP2 locus. In addition, we assessed the association of BP1–BP2 deletions with neuropsychiatric diagnoses, cognitive function and academic achievement. Cases of CVM had an increased prevalence of the deletion compared with controls (0.64%; OR = 1.73 [95% CI 1.08–2.75]; *p* = 0.03), as did those with neuropsychiatric diagnoses (0.68%; OR = 1.84 [95% CI 1.23–2.75]; *p* = 0.004). We conclude that BP1–BP2 deletion moderately increases the risk of the generally milder, but commoner, CVM phenotypes seen in this unselected population, in addition to its previously demonstrated association in case/control studies ascertained for CVM.

## Introduction

**Fig. 1:**
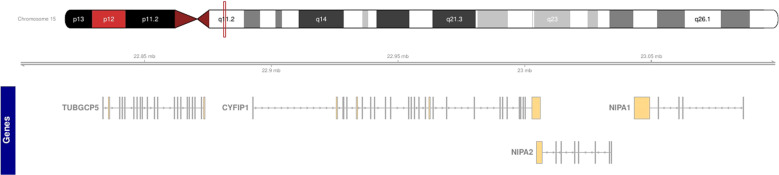
Ideogram of chromosome 15. The location of 15q11.2 and the genes between BP1 and BP2 are indicated.

15q11.2 BP1–BP2 deletions occur between breakpoints 1 and 2 (BP1–BP2; MIM:615656) on the long arm of chromosome 15 and encompass 4 genes (*NIPA1, NIPA2, CYFIP1* and *TUBGCP5*) within a 500-kb region (Fig. 1). The BP1–BP2 region is immediately adjacent to the imprinted regions of chromosome 15 that cause Prader–Willi and Angelman syndromes, but the four genes within the BP1–BP2 region are not imprinted. The clinical and genetic features of this deletion have been recently reviewed by Butler [1]. Individuals with this microdeletion have been described to have an increased risk of a range of neuropsychiatric phenotypes including developmental and speech delays [2–4], autism spectrum disorders [5, 6], attention deficit disorders [7] and schizophrenia [8]. Specific structural and functional consequences of the deletion have been identified in the brain using MRI [9–11]. As with other CNVs causing complex phenotypes (for example 22q11.2 deletion, 1q21.1 duplication), penetrance and expressivity show wide variation. Penetrance of any phenotype with 15q11 BP1–BP2 deletion has been estimated at ~10–12% [2, 12], which is relatively low compared with other CNVs commonly associated with genetic disorders. Taken together, previous studies estimate the prevalence of BP1–BP2 deletion as between 0.19% and 0.47% in apparently healthy controls [2, 8, 12–17].

Genomic deletions are known to be a significant cause of congenital malformations, among which congenital cardiovascular malformations (CVM) have the highest population prevalence of around 9 per 1000 live births [18–24]. We previously identified an excess of heterozygous 15q11.2 BP1–BP2 deletions in a cohort of 2256 patients with a range of non-syndromic CVM phenotypes compared with controls, inferring association of the deletion with CVM risk, albeit with wide confidence intervals (OR = 8.2 [95% CI 1.06–62.98]; *p* = 0.02) [25]. Other studies have found the deletion over-represented in patients selected for CVM [26, 27] and in patients presenting with intellectual disability who also have CVM [2, 7, 28, 29]. However, data are sparse regarding the association of CVM with this deletion in large populations unselected for disease. Since the previous studies focused on CVM tended to recruit patients with more severe, and rarer, CVM phenotypes, information from population-based studies could be useful in estimating risk of commoner, less severe CVM phenotypes.

The UK Biobank (UKB), comprising ~500,000 individuals with genotype data, hospital admission records, baseline cognitive function testing, and a variety of other collected information, provides a large-scale resource for investigating complex genotype–phenotype associations. Here, we determine the prevalence of BP1–BP2 deletions within the UKB cohort and explore its association with CVM; and with neurodevelopmental diagnoses and cognitive function.

## Methods

### CNV calling

B-allele frequency and log2 ratio transformed intensity value files, generated and made available by UKB using the Applied Biosystems UK BiLEVE Axiom and UKB Axiom arrays (*n* = 488,366), were used to generate CNV calls using the PennCNV software [30]. Samples were grouped for CNV calling in batches of ethnically similar samples based on the ethnicity categories defined by UKB (White, Asian or Asian British, Black or Black British, Chinese, Mixed, Other/Unknown). Ethnically ‘white’ samples were further batched for CNV calling into batches of 10,000 samples due to the large number of individuals in this category. PennCNV was run using GCmodel adjustment [31] and, following genome-wide CNV calling, samples with waviness factor < −0.03 or >0.03 were excluded (*n* = 301). Samples containing an excess of CNVs (>40) (*n* = 1401) were also removed at this stage. This threshold intentionally retained samples with a relatively high number of CNVs as global CNV burden has previously been found to contribute to the risk of sporadic CVM [25].

15q11.2 deletions were identified where a heterozygous deletion spanned at least 95% of the four genes involved (*NIPA1, NIPA2, CYFIP1* and *TUBGCP5*) between the outermost array probes covering this region (chr15:22,819,338-23,093,090; GRCh37).

### CVM classification

**Fig. 2:**
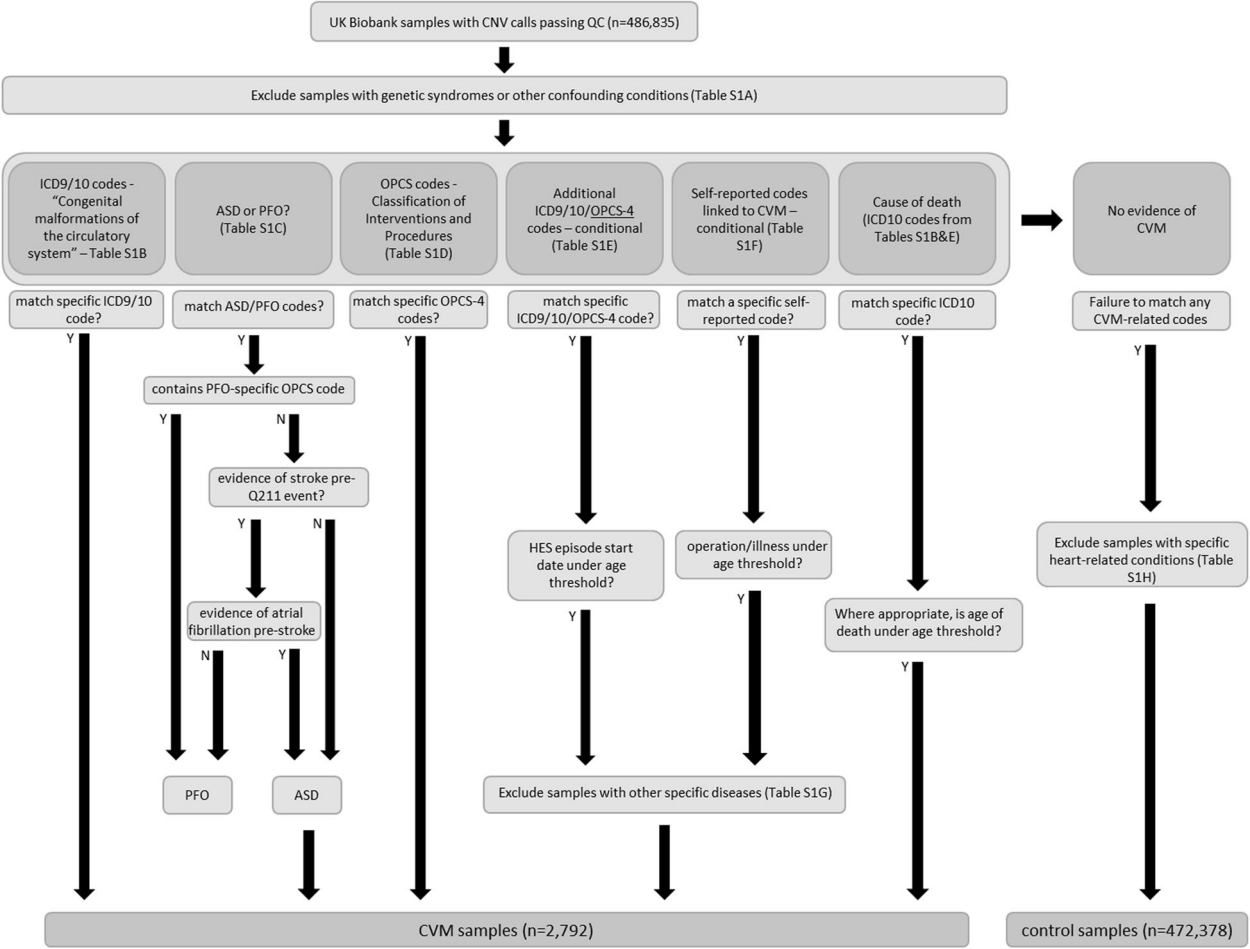
The CVM and control sample classification process on UK Biobank samples using a combination of codes relating to HES data and self-reported information with appropriate thresholds and filtering. Specific codes for each classification step can be found in Supplementary Table 1.

The hospital episode statistics (HES) data available in the UKB contain information on hospital admissions for the cohort. It includes primary and secondary diagnoses in the form of ICD9 and ICD10 codes, as well as details of operations and procedures as recorded through OPCS-4 codes. Using the classification schema shown in Fig. 2, ICD9, ICD10, OPCS-4 and self-reported illness and operation codes were used to classify 2792 UKB samples as having CVM and 472,378 samples as controls, deploying a similar protocol to Saha et al. [32]. To ensure that CVM-classified individuals were non-syndromic we excluded a number of syndromes with possible links to cardiovascular defects from the cohort (codes listed in Supplementary Table 1A). Following this, the initial inclusion criterion for case status was HES evidence of ICD9 or ICD10 codes for ‘congenital malformations of the circulatory system’ (Supplementary Table 1B). Within these, the ICD10 code Q211 can be used for both ‘atrial septal defect’ (ASD) and ‘patent foramen ovale’ (PFO). Since PFO is a normal variant found in up to 25% of the population, we carried out further classification to identify and remove PFOs from the case group. Any sample with a PFO-specific operation code (K165) in addition to a Q211 code was classified as PFO. As PFO is often diagnosed during a stroke workup [33], we also used any diagnosis of stroke without atrial fibrillation prior to Q211 diagnosis as an indicator of PFO rather than ASD. This is because ASD is a common cause of atrial fibrillation, which is a risk factor for stroke, whereas PFO (prior to device closure) is not associated with a significantly increased predisposition to atrial fibrillation (all codes in Supplementary Table 1C). OPCS-4 codes for operations commonly associated with congenital heart defects were also used to classify CVM (Supplementary Table 1D).

In addition to codes covering ‘congenital malformation of the circulatory system’, we used additional codes to identify, amongst others, certain defects of congenital origin presenting in later life (Supplementary Table 1E). Chief among these were codes indicating congenital bicuspid aortic valve (BAV), a condition affecting ~1% of males and ~0.33% of females. We included codes for aortic stenosis and aortic insufficiency as well as associated operational codes. We also used self-reported operation and illness codes (full list is listed in Supplementary Table 1F) indicating ‘aortic valve replacement’, ‘aortic stenosis’ and ‘aortic regurgitation’ as evidence of BAV. An age threshold of diagnosis at younger than 65 years was applied to distinguish individuals with aortic valve disease due to BAV from those with age-related degeneration of a trileaflet aortic valve [34], as previously implemented by Helle et al. [35]. Additional filters were applied to exclude any participants with a diagnosis of other conditions (such as bacterial endocarditis) that might cause non-congenital valve defects. The codes and descriptions of these exclusion diagnoses are listed in Supplementary Table 1G. Individuals with no evidence of CVM phenotypes were classified as controls. Other diagnosed conditions that could potentially have a confounding influence on the classification were excluded from the control set (e.g. PFO-categorised samples, acquired heart defects, samples with evidence of aortic valve disease above the specified age threshold). The full list of excluded codes can be found in Supplementary Table 1H. Finally, deceased UKB participants with or without CVM classifying ICD codes, obtained by UKB from the UK Office for National Statistics, were classified as CVM cases or controls accordingly.

### Neuropsychiatric classification

Classification of neuropsychiatric conditions was determined using ICD10 codes relating to a subset of ‘mental and behavioural disorders’ (Supplementary Table 2A) including schizophrenia, bipolar disorders, autism, mental retardation and various neurodevelopmental disorders to classify case individuals. Individuals with any other code in the mental and behavioural classification (Supplementary Table 2B) were excluded from the control group. This resulted in 3504 case and 483,330 control individuals for neuropsychiatric disorders with CNV calls passing QC thresholds that were then assessed for the presence of BP1–BP2 deletion.

### Cognitive function

We used results from cognitive function tests where the number of participants was at least 10% of the cohort and the test returned numerical data. Tests for ‘reaction time’, ‘numeric memory’, ‘fluid intelligence’ and ‘pairs matching’ met these criteria with varying amounts of missing data (Supplementary Table 3). Our methodology broadly corresponded with previous work in the cohort by others [15, 17]. We removed samples from the whole cohort with ICD10 codes indicating specific diagnoses relating to mental and behavioural disorders previously associated with 15q11.2 deletion (Supplementary Table 2A). In total, there were 3691 samples that matched at least one of these ICD10 codes and were subsequently removed from the cognitive function analysis.

### Statistical analysis

The frequency of BP1–BP2 deletion in cases of CVM, cases of neuropsychiatric disorder, and controls was compared using chi-squared tests. Associations between the deletion and quantitative measures of cognitive function were assessed using different statistical models dependent upon the distribution of the data, and adjusting for age and sex. Linear regression was used for reaction time (removing values greater than five times the standard deviation from the mean). Poisson regression was fitted for fluid intelligence score. For the pairs matching test where values are heavily skewed towards zero, values were converted to a binary variable (0(0), 1(>0)) and logistic regression fitted on the binary outcome. Assessment of the numeric memory test was again assessed with logistic regression by converting the values into a binary variable (0(≤6), 1(>6)). The association between the deletion and the highest qualification achieved was assessed by combining ‘O levels/General Certificate of Secondary Education or equivalent’, ‘Certificate of Secondary Education or equivalent’, ‘National Vocational Qualification or Higher National Diploma or Higher National Certificate or equivalent’, ‘Other professional qualifications’, ‘None of the above’ and ‘Prefer not to answer’ into a single category and treating this as a baseline group. Further details on UK specific qualifications are available at https://www.gov.uk/what-different-qualification-levels-mean/list-of-qualification-levels. The highest qualification achieved per individual was assessed in terms of ‘College/University degrees’ or ‘Advanced levels (A levels)/Advanced Subsidiary levels (AS levels) or equivalent’ and each of these categories compared with the baseline group by fitting a multinomial log-linear model, accounting for age and sex. Fecundity was assessed in males and females separately using Poisson regression fitted to the number of children fathered and number of live births per person, respectively. Outliers were removed if greater than five times the standard deviation from the mean. This excluded samples with >8 children fathered in males and >7 live births in females. An interaction test was performed to confirm the differences between male and female subgroups.

## Results

### Prevalence of BP1–BP2 deletion in non-syndromic CVM

In total, 2792 participants with genetic data available were identified as having non-syndromic CVM and 472,378 were classified as controls (see “Methods”). Table 1 shows a list of the most common CVM diagnoses and associated sample numbers in UKB based upon compiling relevant ICD9, ICD10, OPCS-4 and self-reported codes. A full list of these codes along with diagnoses and associated number of individuals can be found in Supplementary Table 4. Among the participants with CVM, the most common diagnoses relate to the presence of BAV.

**Table 1:** The most common CVM diagnoses in UK Biobank.

CVM diagnosis	Cases
Aortic valve replacement (<65 years)	896
Aortic stenosis (<65 years)	841
Aortic insufficiency (<65 years)	594
Atrial septal defect	409
Malformation of vascular system	369
Heart surgery (<18 years)	212
Congenital heart disease—unspecified	110
Ventricular septal defect	95
Cardiac septum defect—unspecified	90
Pulmonary insufficiency	50
Pulmonary stenosis	45
Aortic valve issue—unspecified (<65 years)	36
Coarctation of aorta	30
Pulmonary artery issue—unspecified	29
Patent ductus arteriosus	28
Aortic defect—unspecified	23
Dextrocardia	19
Heart block	17
Tetralogy of Fallot	17
Aortic atresia	13
Ebstein’s anomaly	12
Pulmonary valve defect	12
Atrioventricular septal defect	11
Anomalous pulmonary venous return	10

In total, 1832 BP1–BP2 deletions were identified in UKB participants with genotyping data available. The CVM and control groups accounted for a total of 1787 of the samples with BP1–BP2 deletion, with 45 deletions present in participants who were excluded according to the criteria outlined in “Methods”. Of the 2792 non-syndromic CVM samples, 18 carried the BP1–BP2 deletion resulting in an estimated prevalence of 0.64% (Table 2A). The specific phenotypes and classifying codes of these cases are summarised in Supplementary Table 5A. Of 472,378 control samples, 1769 carried the BP1–BP2 deletion resulting in a prevalence of 0.38% (OR = 1.73 [95% CI 1.08–2.75]; *p* = 0.03). In keeping with previous observations, no participant was homozygous for the BP1–BP2 deletion.

**Table 2:** (A) The number and prevalence of BP1–BP2 deletions in non-syndromic CVM cases compared with controls in UK Biobank. (B) The number and prevalence of BP1–BP2 deletions in neuropsychiatric cases compared to controls in UK Biobank.

	BP1–BP2 del	No BP1–BP2 del	Prevalence
**A**			
Non-CVM controls	1769	470,611	0.38%
All non-syndromic CVM samples	18	2774	0.64%
**B**			
Non-neuropsychiatric controls	1808	481,522	0.37%
Neuropsychiatric cases	24	3480	0.68%

Two of the eighteen CVM case samples carrying the BP1–BP2 deletion also had neuropsychiatric diagnoses. Those individuals had cardiovascular diagnoses of ASD and aortic valve disease/replacement; and neuropsychiatric diagnoses of bipolar disorder and scholastic developmental disorder, respectively.

### Prevalence of BP1–BP2 deletion in neuropsychiatric disorder cases

Three thousand five hundred and four individuals were found to have a relevant diagnosis in the HES data. Of these, 24 carried the deletion, a prevalence of 0.68%, which was significantly different from the control group (OR = 1.84 [95% CI 1.23–2.75]; *p* = 0.004) (Table 2B). The specific diagnoses of these 24 individuals can be found in Supplementary Table 5B.

### BP1–BP2 deletions and cognitive function

**Fig. 3:**
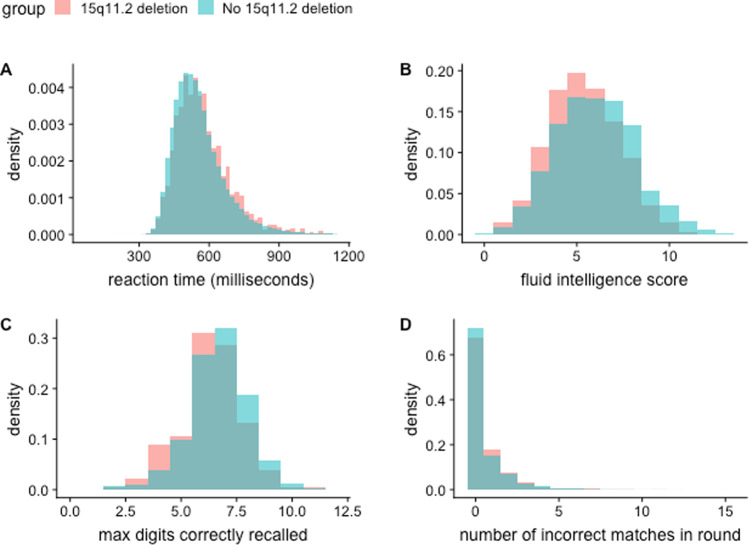
Cognitive function test differences between individuals carrying a 15q11.2 deletion and those without. **a** Reaction time test. **b** Fluid intelligence score. **c** Numeric memory test. **d**. Pairs matching test.

After removing participants with relevant neuropsychiatric disorders, 483,330 samples remained. Of these, 1808 carried the BP1–BP2 deletion. Figure 3 shows the differences between the two groups in terms of the scores in four different cognitive function tests. Table 3 summarises the results of these tests and indicates differences in performance between individuals with and without BP1–BP2 deletion, after adjustment for participants' age and sex.

**Table 3:** Summary of the cognitive function tests in the BP1–BP2 deletion group and non-carriers.

	15q11.2 del				No 15q11.2 del				
Test	*N*	Mean	Median	SE	*N*	Mean	Median	SE	*p* value
Reaction time (ms)	1781	574.60	551.0	2.789	475,318	556.80	536.0	0.160	1.38E−13
Fluid intelligence score	556	5.40	5.0	0.080	158,373	5.99	6.0	0.005	8.02E−09
Numeric memory	188	6.07	6.0	0.140	50,158	6.48	7.0	0.008	9.00E−06
Pairs matching	1808	0.61	0.0	0.030	480,590	0.54	0.0	0.002	2.59E−03

### BP1–BP2 deletions and academic achievement

Carriers of the BP1–BP2 deletion were compared with non-carriers in terms of the highest academic qualifications that they had achieved. Table 4 shows the number of each cohort with ‘College/University degree’ and ‘A levels/AS levels’ as their highest qualification compared with a combined ‘Other categories’ group. We observed lower proportions of deletion carriers obtaining college/university degrees and Advanced and AS level qualifications (typically taken at age 17–18 and required for university or college entrance). Overall, 32.8% of participants without BP1–BP2 deletion had attained a College/University degree, whereas 22.8% of deletion carriers had done so (OR = 0.57 [95% CI 0.51–0.64]; *p* = 5.60E−22). Similarly, a lower proportion of participants with BP1–BP2 deletion had attained A/AS levels as their highest educational qualification than participants without the deletion (OR = 0.76 [95% CI 0.65–0.88]; *p* = 4.12E−04).

**Table 4:** Academic qualifications achieved by carriers of BP1–BP2 deletion in comparison with non-carriers.

	BP1–BP2 deletion	No BP1–BP2 deletion		
Highest qualification achieved	Frequency	Frequency	Odds	*p* value
College/University degree	414	157,988	0.57	5.60E−22
A levels/AS levels	185	54,077	0.76	4.12E−04
Other categories	1209	268,962	–	–

‘College/University degree’ and ‘A levels/AS levels’ highest qualification attainment groups are each compared with a combined ‘Other categories’ group. Estimated odds ratios reflect the chance of the BP1–BP2 deletion group achieving the qualification compared with non-carriers.

### BP1–BP2 deletions and fecundity

**Fig. 4:**
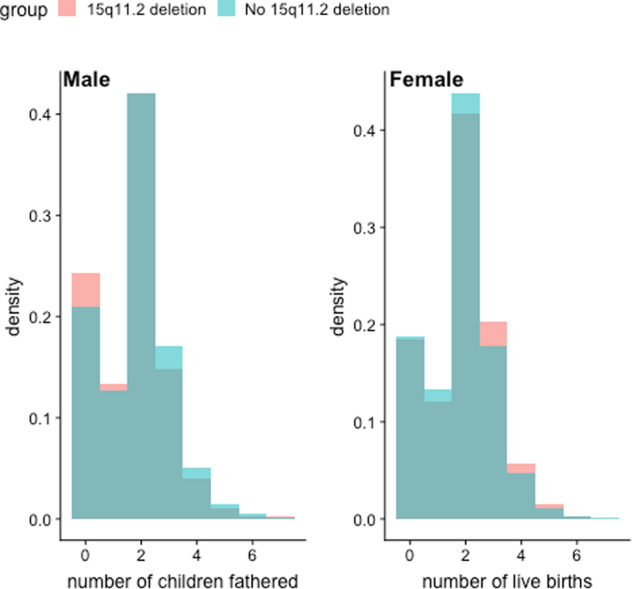
Fecundity differences between individuals carrying a 15q11.2 deletion those without. Fecundity is measured as self-reported number of children fathered (by males) and number of live births (by females).

Fecundity was assessed in those individuals with and without the BP1–BP2 deletion. Self-reported number of children fathered (for males) and number of live births (for females) in each cohort are shown in Fig. 4. A difference was identified in males between those individuals carrying the BP1–BP2 deletion and those without (mean number of children fathered = 1.66 and 1.8, respectively; *p* = 0.00175). This difference was not evident in females (mean number of live births = 1.88 and 1.82 in BP1–BP2 deletion carriers and non-carriers, respectively; *p* = 0.18). A test for interaction confirmed the differences between the male and female subgroups (*p*-interaction = 5.46E−04).

## Discussion

The 15q11.2 (BP1–BP2) deletion (sometimes referred to as the Burnside-Butler syndrome susceptibility locus) has previously been associated with phenotypes including developmental delay, autism, schizophrenia and CVM; the great majority of the evidence regarding the deletion thus far originates from cohorts specifically selected for one or other of those phenotypes. We estimated the prevalence of the deletion and its associations with CVM and other diagnoses in the UKB cohort. We confirmed the association of the deletion with CVM (OR = 1.73 [95% CI 1.08–2.75]; *p* = 0.03) and, in broad agreement with recent findings from others [17], with neuropsychiatric disorders (OR = 1.84 [95% CI 1.23–2.75]; *p* = 0.0043), measures of cognitive function, academic achievement and fecundity.

We confirmed the association of BP1–BP2 deletion with CVM, which has been observed in some but not all previous studies [25–27, 36]. The first study linking BP1–BP2 deletions with non-syndromic CVM showed an odds ratio of 8.2 [95% CI 1.06–62.98]; the point estimate for the risk associated with the deletion was much less in this study, but still falling within the 95% confidence interval of the estimate from the first study. A number of CVM cases contributing to the hypothesis-generating study and to the present results in the UKB are broadly comparable. However, there are major differences between the range of phenotypes in the hypothesis-generating study (and other studies ascertaining principally on CVM) and UKB, less severe conditions such as BAV predominating in UKB. The results tend to suggest the deletion confers a higher risk of more severe and less common CVM conditions, by comparison with less severe, commoner CVM conditions, but the present study cannot be conclusive on this issue. Larger studies of specific CVM phenotypic groups will be required to resolve this.

Of note, the prevalence of CVM in UKB, excluding BAV related conditions, was considerably lower than in birth prevalence data from the UK and Europe [21]; this would be consistent with the known ‘healthy cohort bias’ in UKB. The prevalence of definite or inferred BAV was also lower than anticipated; it is most likely that not all members of the UKB cohort would have reached an age where BAV had manifested clinically, so some BAV cases will have been misclassified as controls. However, there is no evidence that such misclassification would be differential between 15q11.2 deletion carriers and non-carriers. In future studies, availability of electronic health records from the General Practitioners of UKB participants, may mitigate any misclassification bias in respect of CVMs.

A recent meta-analysis of phenotypic associations of the 15q11.2 deletion substituted the prevalence of the deletion in UKB healthy controls of 0.36% for the prevalences actually observed in previous studies of CVM, and concluded that there was no evidence for enrichment for the deletion in CVM cases [36]. We suggest that such between-study comparison of prevalences determined on different assay platforms may not be straightforward to interpret. Also in that paper, the reciprocal 15q11.2 duplication was used as a pseudo-control in some analyses. No excess of CVM cases was observed among individuals carrying a deletion compared with those carrying a duplication, interpreted as evidence of no association. But, no previous study has conclusively determined that the reciprocal duplication carries no increased risk of CVM; it is known for certain other deletion/duplication syndromes (such as 22q11.2 and 1q21.1) that both deletion and duplication may increase CVM risk.

Estimates of the prevalence of BP1–BP2 deletion have varied widely in the literature. The first large-scale population study, by Stefansson et al. [16], showed a general population prevalence of 0.23% (95% CI 0.21–0.26) in 101,655 Icelandic subjects of whom 241 carried the BP1–BP2 deletion. Given differences in recruitment strategies, genotyping methodologies and populations, direct comparison between this figure and our estimate of 0.37% (95% CI 0.36–0.39) is not straightforward; however, they are broadly consistent with each other. The previous study by Stefansson had also shown nominally reduced fecundity among 172 carriers of the BP1–BP2 deletion who were over 45 years of age. In UKB, we observed reduced fecundity in males carrying this deletion (*p* = 0.00175) (Fig. 4), supporting these findings in a much larger cohort. Stefansson et al. concluded that the neuropsychiatric manifestations of the BP1–BP2 deletion in carriers without schizophrenia were most evident on dyslexia and dyscalculia [10, 16]. The present study had a less extensive range of cognitive function tests, but with larger numbers of deletion carriers, and in agreement with recent observations [17], we observed a more general effect on cognitive function among carriers who had not been diagnosed with a neuropsychiatric disorder. In addition, we showed sizeable associations between the deletion and lower educational attainment.

The clinical and genetic aspects of BP1–BP2 deletion have been recently reviewed [1]. Four genes, *TUBGCP5, CYFIP1, NIPA1* and *NIPA2*, are located in the deleted region. The effects of hemizygosity at each individual gene in the region have not been systematically described in mouse models, nor has any mouse model of the entire deletion been reported as yet. Therefore, the mechanisms whereby the deletion causes its associated phenotypes, and the interaction between hemizygosity for the region and other genetic and environmental factors to result in the wide variety of phenotypes described, remain to be elucidated.

Regarding the classification of the deletion for the purposes of clinical genetics and genetic counselling, we tend to agree with Jønch et al. that the deletion should be classified as “pathogenic, of mild effect size” [36]. The effect on CVM is not sufficient to justify genetic testing in children presenting with CVM as their primary diagnosis, and our conclusions regarding neuropsychiatric manifestations of the deletion are in agreement both with that previous study, and others in the UKB cohort [17]. More generally, the highly variable severity of outcomes with the 15q11.2 deletion, as with other CNVs associated with neurodevelopmental disorders and organ system malformations, suggests the existence of important modifying factors, which may be both genetic and environmental; further investigations directed at identifying these factors will be an important priority for future research.

## Supplementary information

Supplementary Table Legends

Supplementary Table 1

Supplementary Table 2

Supplementary Table 3

Supplementary Table 4

Supplementary Table 5

## Data Availability

The 15q11.2 deletions in the UKB cohort are available through the European Variation Archive (https://www.ebi.ac.uk/eva/; Project: PRJEB35772; Analyses: ERZ1231712).
