# Supplementary material for: Association of congenital cardiovascular malformation and neuropsychiatric phenotypes with 15q11.2 (BP1–BP2) deletion in the UK Biobank
Source: Eur J Hum Genet. 2020 Apr 23;28(9):1265–73. doi: 10.1038/s41431-020-0626-8 (PMC7608352; doi:10.1038/s41431-020-0626-8)
Supplement: Supplementary file 1 — Supplementary Table Legends [file 41431_2020_626_MOESM1_ESM.docx]

**Supplementary material**

**Supplementary Table 1.** Lists of ICD9, ICD10 and OPCS4 codes used as inclusion and exclusion criteria for classifying the UK Biobank cohort as non-syndromic CVM samples or controls.

**Supplementary Table 2**. (**A)** List of ICD10 codes used to classify individuals as having had phenotypically relevant neuropsychiatric disorders. (**B)** Additional ICD10 codes for ‘mental or behavioural disorders’ that were used to exclude individuals from the control group for neuropsychiatric disorders.

**Supplementary Table 3**. Amount of ‘missing’ data in the cognitive function tests.

**Supplementary Table 4**. A list of CVM-classifying codes and the number of samples in UK Biobank with these classifications.

**Supplementary Table 5**. Phenotypes of UK Biobank participants classified as having (**A)** CVM diagnoses and a BP-BP2 deletion and (**B**) neuropsychiatric diagnoses and a BP1-BP2 deletion.
